# Multimodal Approaches in the Management of Temporomandibular Disorders: A Narrative Review

**DOI:** 10.3390/jcm14124326

**Published:** 2025-06-17

**Authors:** Izabela Dąbkowska, Lena Sobiech, Agata Czępińska, Adam Bęben, Karolina Turżańska, Piotr Gawda

**Affiliations:** 1 Interdisciplinary Scientific Group of Sports Medicine, Department of Sports Medicine, Medical University of Lublin, 20-093 Lublin, Poland; 2 Student of Dentistry, Medical University of Gdańsk, ul. M. Skłodowskiej-Curie 3a, 80-210 Gdańsk, Poland; 3 Department of Sports Medicine, Medical University of Lublin, 20-093 Lublin, Poland; 4 Department of Dental Prosthetics, Medical University of Gdańsk, ul. M. Skłodowskiej-Curie 3a, 80-210 Gdańsk, Poland; 5 Department of Rehabilitation and Orthopedics of Lublin, Medical University of Lublin, 20-093 Lublin, Poland

**Keywords:** TMD, TMJ, disorders, manual therapy, physical therapy, dry needling, botulinum toxin, Botox, splint, psychotherapy

## Abstract

**Background/Objectives:** Temporomandibular disorders (TMDs) are the most common cause of non-dental pain in the orofacial region. Due to the complex and multifactorial nature of TMD, a multidisciplinary approach is often required. The objective of this narrative review is to evaluate the effectiveness of multimodal therapies in the management of TMD. **Methods:** A literature search was performed using a combination of keywords: “TMD”, “TMJ”, “disorders”, “manual therapy”, “physical therapy”, “dry needling”, “botulinum toxin”, “Botox”, “splint”, and “psychotherapy”. The search was conducted in the PubMed, Google Scholar, and Scopus databases, focusing on studies involving human subjects. **Results:** The included studies reported that the use of multimodal approaches—such as physiotherapy, botulinum toxin injections, occlusal splints, and/or psychotherapy—led to symptom improvement or complete resolution in patients with TMD. **Conclusions:** Temporomandibular disorders are complex conditions with a multifactorial etiology involving both somatic and psychological components. Given the wide range of symptoms and the functional connections of the temporomandibular joint with the nervous, muscular, and skeletal systems—including the cervical spine—effective treatment of TMD requires a multidisciplinary strategy.

## 1. Introduction

The temporomandibular joint (TMJ) is a bilateral synovial joint located on either side of the craniomandibular complex [1,2]. It consists of the mandibular fossa, the articular eminence, and the condylar process of the mandible, separated by an articular disc. The TMJ plays an essential role in functions such as swallowing, chewing, speech, and involuntary movements including yawning, teeth grinding, and clenching [1,3,4].

Temporomandibular disorders (TMDs) are becoming increasingly prevalent [5,6]. The term refers to pain and dysfunction of the TMJ and related structures, most commonly involving the masticatory muscles [7,8]. Clinical symptoms may include headaches, restricted joint mobility, hypertrophy and pain in the masticatory muscles, joint inflammation, and audible sounds such as clicking or crepitus during mandibular movements [9].

The etiology of TMD remains unclear and is considered multifactorial, involving biomechanical, neuromuscular, biological, and biopsychosocial components [5,10,11,12]. Occlusal overload and parafunctions such as bruxism are key biomechanical contributors. Elevated levels of estrogen have also been proposed as biological factors influencing TMJ function, particularly in women. Estrogen levels have been shown to be associated with pain modulation in the temporomandibular joint and the entire orofacial region [13,14]. Among biopsychosocial factors, stress, anxiety, and depression are frequently reported [15,16].

Conditions associated with TMD include bruxism, capsulitis, and intra-articular disc abnormalities [1,17]. Dental occlusion and bruxism are commonly diagnosed as comorbidities. The co-occurrence of TMD and bruxism is thought to be influenced by overlapping genetic, hormonal, ethnic, and geographical factors [12,18]. Zieliński et al. suggest that the co-occurrence of bruxism and TMD in the global population is approximately 17% [12]. Additionally, the authors observed that a 1% increase in the proportion of women in the study group was associated with a 4.4% increase in the likelihood of TMD and bruxism co-occurrence [12], highlighting the influence of sex as a contributing factor in TMD [13,14,19,20].

TMD is the most common cause of non-dental pain in the orofacial region [21]. The global prevalence of TMD is estimated at around 34%, although this figure varies by region and population group [10]. For instance, the occurrence of TMD was notably greater in South America, reaching 47%, in contrast to 33% in Asia and 29% in Europe [10]. Chronic pain is the most frequent reason why patients seek professional help [22,23].

Numerous studies suggest a link between TMD and cervical spine dysfunction [24,25,26,27,28]. This association is believed to result from the neuroanatomical convergence of nociceptive inputs from the trigeminal and cervical nerves. The topographical organization of the spinal trigeminal nucleus allows bidirectional information transfer between these systems. Consequently, stimulation of structures innervated by the trigeminal nerve can lead to cervical pain, and vice versa [24,25,26,27,28]. It shows the complexity of the factors that may influence the TMJ.

There is no universally effective treatment for TMD [2,29,30]. Due to its multifactorial nature, an interdisciplinary approach is essential. Effective management requires the collaboration of dentists, physiotherapists, and mental health professionals, given the well-established relationship between TMD and psychological stress [6,7,16,31,32]. Each case should be approached individually, with a comprehensive diagnostic process to identify the primary contributing factors [33].

Currently, conservative treatment is recommended as the first-line approach for managing TMD [34]. This includes physiotherapy involving manual techniques targeting the joint and surrounding soft tissues, muscle relaxation or strengthening exercises, and physical therapy modalities [35]. Occlusal splints are commonly used to stabilize the mandible and reduce muscle tension [36,37]. For moderate to severe pain, pharmacological interventions such as non-steroidal anti-inflammatory drugs (NSAIDs), analgesics, and muscle relaxants have shown clinical effectiveness [38,39,40].

In cases where conservative therapies fail to achieve satisfactory outcomes, more invasive interventions may be considered. Intra-articular corticosteroid injections are used to reduce inflammation, and in cases of persistent muscular hyperactivity, botulinum toxin type A (BoNT-A) has been applied. BoNT-A is approved by the FDA for the treatment of various pain conditions, including certain forms of dystonia [41,42].

The purpose of this narrative review is to evaluate the effectiveness of a multimodal, multidisciplinary approach in the treatment of temporomandibular disorders.

## 2. Materials and Methods

A literature search for relevant papers indexed in the literature from 2020 to 2025 was conducted using the PubMed, Scopus, and Google Scholar databases. This time frame was chosen because it covers the period directly following the outbreak of the COVID-19 pandemic. The pandemic had a significant impact on both the mental and physical health of the population, increasing levels of stress and anxiety, and altering many people’s lifestyles—all of which are closely linked to the development and exacerbation of TMD symptoms [43,44,45]. The intensification of these factors may also have contributed to the advancement of research and therapeutic approaches aimed at improving the effectiveness of treatment methods [46,47,48].

Three researchers reviewed the databases from 1 March to 1 May 2025. The search was conducted by two independent researchers, and all discrepancies were resolved by a third investigator. In our paper, we included clinical trials, pilot studies, and prospective studies concerning the impact of TMD disorders, physical therapy, manual therapy, botulin toxin BTX-A, and splints. The methodology for study selection was adapted from [10,18].

Keywords included “TMD”, “TMJ”, “disorders”, “manual therapy”, “physical therapy”, “dry needling”, “botulinum toxin”, “Botox”, “splint”, and “psychotherapy”. The keywords were searched with MeSH terms. Only studies published in English were considered to maintain clarity and accuracy in data interpretation The authors reviewed selected articles according to topic relevance. The reference lists of selected manuscripts were also revised. Relevant titles identified during this process led to further examination of the corresponding articles. If these articles provided additional pertinent information, they were subsequently included in the review. The inclusion criteria are listed in Table 1.

## 3. Treatment of Temporomandibular Joint Disorders

### 3.1. TMD Disorders and Physiotherapy Treatment

#### 3.1.1. Manual Therapy and Joint Mobilization

There is no single gold standard in the treatment of TMD [2,29,30], and the literature increasingly supports the use of multimodal therapeutic approaches [10,48,49]. Among conservative interventions, physiotherapy plays a central role, particularly through manual therapy, exercise, and adjunct physical modalities.

One frequently applied technique in the management of TMJ dysfunction is joint mobilization, particularly the Maitland mobilization method. This technique has been reported to restore normal function in both joints and surrounding muscles [50,51]. In a study by Nambi and Abdelbasset, the effectiveness of a 4-week Maitland mobilization program was compared to a home-based training regimen in patients with TMJ dysfunction following bilateral cervicofacial burns [50]. The group receiving Maitland mobilizations demonstrated significantly greater improvements in mouth opening, pain intensity, functional limitations, kinesiophobia, sleep quality, and overall quality of life. These findings highlight the benefit of targeted manual therapy, particularly in cases where scar-related contractures contribute to TMJ dysfunction [6,52].

Complementary findings were reported by Barone et al., who assessed the immediate effects of a single session of rhythmic TMJ mobilization. Results showed statistically significant improvements in pain intensity, pressure pain threshold, and mandibular opening, suggesting that even short-term interventions can provide measurable relief [53].

#### 3.1.2. Therapeutic Exercise and Yoga-Based Interventions

Therapeutic exercises represent another cornerstone in the physiotherapeutic management of TMD. Gębska et al. emphasized their simplicity and safety, especially in patients with myogenic TMD [54].

Furthermore, yoga-based exercise programs have emerged as an alternative therapeutic approach. Long-term yoga practice has been shown to modulate pain centers in the brain and improve pain tolerance [55]. In a study by Atilgan et al., yoga sessions incorporating breathing techniques, relaxation, and physical postures (asanas) significantly reduced TMJ and neck pain, improved range of motion, and alleviated depressive symptoms in women with myofascial TMD [56].

Vassis et al. demonstrated that jaw-targeted exercises in children with juvenile idiopathic arthritis—a rheumatic disease that often affects the facial region—led to a reduction in pain frequency and intensity, as well as improvements in jaw function, including maximum mouth opening, laterotrusion, and protrusion [57].

#### 3.1.3. Cervical Spine Integration and Trigeminocervical Complex

The interconnection between the cervical spine and TMJ is another important consideration. Anatomically, biomechanically, and neurophysiologically, the cranio-mandibular and upper cervical regions function as an integrated system, linked via the trigeminocervical complex, which facilitates nociceptive signal transmission [26,58,59,60]

Studies have shown impaired neck muscle function and structural deficits in patients with TMD [61,62]. Rezaie et al. found that integrating manual therapy targeting both the upper cervical spine and TMJ into standard care resulted in superior outcomes compared to routine treatment alone [63].

Similarly, Crăciun et al. reported that a 3-month physiotherapy protocol targeting the cervical and temporomandibular regions significantly reduced pain and improved Jaw Functional Limitation Scale and Neck Disability Index scores [64]. Further supporting this, Oliveira-Souza neck et al. demonstrated that motor control training for neck muscles was significantly more effective than the placebo in improving jaw pain and function [65].

#### 3.1.4. Dry Needling and Combined Interventions

Another promising modality is dry needling, which targets myofascial trigger points to disrupt pathological impulses and improve local circulation [66,67]

Dib-Zakkour et al. found that deep needling of the masseter muscles resulted in reduced pain, decreased muscle activity, improved mandibular positioning and motion symmetry, and increased maximum mouth opening compared to a placebo group [66]. Moreover, Dunning et al. demonstrated that combining dry needling with cervical spinal manipulation produced significantly greater improvements in pain relief and pain-free mouth opening than standard treatments such as NSAIDs, occlusal splints, and TMJ mobilization [68].

#### 3.1.5. Myofascial Techniques and Muscle Relaxation

Manual techniques such as post-isometric relaxation (PIR) and myofascial release are also effective in TMD with a predominantly muscular component.

Research by Urbański et al. showed that both approaches significantly reduced muscle tension and bioelectrical activity in the masseter and anterior temporalis muscles, as well as lowering pain intensity [69].

Tariq and co-authors published a paper, the main aim of which was to compare the effects of massage therapy alone and massage therapy combined with post-isometric relaxation exercises in patients with TMD in terms of pain and maximum mouth opening. Group A received conventional treatment including massage and therapeutic exercises consecutively for 2 weeks. Group B (*n* = 23) received conventional treatment combined with a post-isometric relaxation technique. Both groups showed significant improvements in pain and MMO scores after treatment. However, the group that received a massage with isometric relaxation exercises showed significantly better results compared to the group of participants who received a massage alone. This suggests that the combination of PIR and a massage was more effective in treating TMD than a massage alone [70].

A study by Kuć and co-authors on 50 patients diagnosed with myofascial pain with radiation showed that soft tissue mobilization leads to a significant reduction in masticatory muscle activity, suggesting the effectiveness of this therapy in muscle relaxation in TMD patients [71]. On the other hand, Javed and co-authors when studying the effects of the post-isometric relaxation technique and Bowen therapy on pain, range of motion, and functional activity in patients with temporomandibular joint disorders found that post-isometric relaxation was more effective in terms of pain, range of motion of opening the mouth, lateral deviation, and functional activity of patients with temporomandibular joint disorders [72].

Another randomized clinical trial involving 72 patients found that the trigger point release technique (PRT) significantly reduced pain and improved function compared to a placebo. The effects persisted for at least three months after the therapy was completed [73].

#### 3.1.6. Physical Modalities

Recently, interest has also grown in the application of physical modalities such as extremely low-frequency magnetic field (ELF-MF) therapy, either alone or in combination with LED light therapy.

Kubala et al. reported that all intervention groups experienced a significant reduction in pain perception on the VAS, indicating the potential value of these adjunct techniques in managing masticatory muscle hypertonia [74]. Physical therapy is also becoming a valuable tool in aiding recovery from invasive treatments for temporomandibular joint disorders.

Frisca and co-authors demonstrated in their study that pulsed electromagnetic fields (PEMFs) can accelerate the reduction in swelling and pain in patients undergoing orthognathic surgery in the maxillofacial surgery department. The results of their study suggest that it is worth using PEMF to accelerate postoperative recovery [75].

#### 3.1.7. Patient Education and Multimodal Strategies

Lastly, patient education is a fundamental component of physiotherapeutic interventions. Evidence suggests that combining manual and physical therapy with education yields better short-term outcomes than home exercise or education alone [76].

The Aguiar et al. study shows that adding a new individualized educational intervention on pain science improved disability in individuals receiving manual therapy and exercise-based care for TMD [77]. Counseling, including education about etiological factors, avoidance of parafunctional and sleep habits, and dietary advice, significantly reduced pain intensity and improved oral health-related quality of life in patients with TMD. Compared with the control group, patients who received counseling showed a greater and consistent improvement in both pain and quality of life throughout the follow-up period [78].

In the study by Liu et al., the authors conclude that patient education and targeted treatment contribute to the improvement of oral behaviors (OBs). Factors influencing this improvement included patient age, level of education, and the initial severity of their OBs [79]. Education included an explanation of the anatomy and functioning of the temporomandibular joint, teaching the resting position for the joint, and posture correction. Patients also received a list of individual recommendations (Table 2).

### 3.2. Occlusal Splints of TMD: Mechanisms and Clinical Applications

Occlusal splints have gained wide acceptance as a conservative, non-invasive option for managing TMD. Their therapeutic potential lies in the mechanical redistribution of occlusal forces across the dental arch, which helps to mitigate excessive tooth wear, relieve muscle tension, and foster symmetrical masticatory muscle activity. This mechanical equilibrium plays a crucial protective role for the TMJ, minimizing dysfunctional loading that can predispose to disc displacement and degenerative changes. Moreover, by establishing a stable occlusal relationship, splints contribute to neuromuscular balance, which is essential for long-term symptom relief [37].

Recent studies have begun to unravel the neuromuscular implications of splint therapy. For example, in their research, Gupta et al. examined the electromyographic (EMG [80]) activity of both masticatory and cervical muscles, including the masseter, temporalis, sternocleidomastoid, and digastric muscles. TMD patients were observed to exhibit diminished EMG activity at rest and during function, a possible indicator of muscular inefficiency or dysfunction. Following a three-month regimen of wearing a flat upper stabilization splint, patients demonstrated a noticeable increase in both resting and functional EMG activity. In addition, improved symmetry in muscle activation suggested a reestablishment of coordinated muscle function. These results underscore the neuromuscular benefits of occlusal splints beyond simple joint stabilization [81].

Given the multifactorial nature of TMD, combination therapies have gained prominence. A study by Bachani et al. compared the outcomes of patients treated with occlusal splints alone to those receiving additional therapeutic exercises. While both groups experienced symptomatic relief by the third week, the group undergoing combination therapy showed earlier improvements in pain reduction. The splints facilitated condylar repositioning and occlusal contact balance, while the exercises likely addressed muscular tightness and improved joint function. These findings suggest that a synergistic approach—mechanical and functional—can accelerate recovery and improve overall outcomes, especially in patients with moderate to severe TMD symptoms [82].

Another layer of complexity in TMD management involves patient engagement with therapy protocols. Wänman et al. investigated this by assigning patients to either splint therapy, unsupervised home exercises, or supervised physiotherapeutic training. Interestingly, the home exercise group exhibited the highest dropout rate, implying that motivation and compliance are critical factors in therapeutic success. While joint sound reduction was comparable across all groups, supervised exercise seemed to offer additional benefits in terms of general well-being and patient satisfaction. This reinforces the idea that supportive, structured therapy environments may foster greater long-term adherence and psychosocial resilience in TMD patients [83] (Table 3).

### 3.3. Botulinum Toxin Treatment of TMD

While occlusal splints provide a mechanical and neuromuscular approach to the management of temporomandibular disorders, botulinum toxin type A (BTX-A) has emerged as a promising biochemical intervention targeting muscular hyperactivity and pain. TMD often presents with chronic myofascial discomfort, joint noises, and functional limitations of the masticatory apparatus [84]. BTX-A, a neurotoxin known for its ability to block presynaptic acetylcholine release at the neuromuscular junction and to inactivate calcium channels at nerve endings, leads to temporary muscle paralysis [85]. This mechanism offers a targeted approach to alleviating tension and pain in overactive masticatory muscles.

Clinical evidence consistently supports the efficacy of BTX-A in reducing myofascial pain. In a randomized controlled trial, De la Torre Canales et al. conducted a longitudinal study on female patients suffering from persistent myofascial TMD, administering BTX-A in varying doses to the masseter and temporalis muscles [86]. Over a 72-month follow-up, patients reported sustained reductions in pain intensity as measured by the Visual Analog Scale (VAS), and objective increases in pain pressure threshold (PPT) values. Interestingly, although a short-term reduction in muscle thickness was observed via ultrasonography, this effect was reversible, with no significant long-term change at six years [87]. These findings underscore both the durability of the analgesic effects and the reversibility of muscular atrophy, addressing concerns about long-term muscle degradation. Nonetheless, some studies have questioned the consistency of these outcomes across patient populations, with varying reports of efficacy depending on the BTX-A dosage, injection technique, and baseline severity of symptoms.

Beyond pain reduction, BTX-A therapy has demonstrated benefits in relieving secondary symptoms such as headache. Several studies suggest that TMD-related myofascial trigger points (MTrPs) may act as sources of referred pain, contributing to tension-type or myogenic headaches [88]. In this context, BTX-A appears to exert therapeutic effects through both peripheral desensitization and central modulation of nociceptive pathways. So Ra Kim and José A. Blanco-Rueda et al., in their pilot study, found notable reductions in headache frequency and intensity following BTX-A injections in tender masticatory regions [84,89]. In fact, Blanco-Rueda’s group found headache relief in over 70% of patients at a six-week follow-up, supporting a close neurophysiological link between orofacial myofascial pain and headache syndromes [84].

However, these findings should be interpreted with caution, as placebo-controlled trials remain limited, and the duration of headache relief is often transient.

An emerging advancement in BTX-A administration is the use of ultrasound (US) guidance to increase precision and reduce complications. Real-time US imaging enables accurate localization of the target muscle, particularly the masseter, and helps avoid surrounding anatomical structures. Visualization of all three heads of the masseter muscle allows for an even distribution of the toxin, improving therapeutic outcomes while minimizing risks such as asymmetry or unintentional diffusion. Furthermore, ultrasound guidance supports the adjustment of the needle depth based on the soft tissue thickness and enables a quantitative assessment of the treatment response by measuring changes in muscle thickness pre- and post-injection. This technique is particularly valuable for optimizing efficacy and ensuring a reproducible placement in both clinical and research settings [90].

When examining TMJ-related symptoms, such as joint clicking, the literature reveals mixed outcomes. Rezazadeh et al. reported no statistically significant improvement in TMJ sounds following extraoral BTX-A injections into the lateral pterygoid muscle [86]. Conversely, Blanco-Rueda et al. observed a subjective improvement in joint noise in 75% of cases after a combined intra-articular and intramuscular BTX-A protocol [84]. This discrepancy may reflect differences in injection sites, dosages, or evaluation metrics, indicating the need for further standardized investigations. Moreover, while some patients report auditory symptom relief, objective verification through imaging or acoustic assessment is often lacking.

In terms of functional improvements, botulinum toxin has also shown promise in enhancing mandibular mobility. In a randomized controlled trial, Rezazadeh et al. observed increased lateral jaw movement following BTX-A injection into the lateral pterygoid [86], while other studies, including those by De la Torre Canales and Hosgor, confirmed increased maximum mouth opening (MMO) post-treatment [91,92]. Notably, improvements were seen across low-, medium-, and high-dosage groups, suggesting that the functional gains may not be strictly dose-dependent. After 180 days, all treated groups demonstrated a statistically significant enhancement in range of motion compared to the placebo [92] (Table 4).

Despite these promising findings, BTX-A should be considered an adjunct rather than a first-line treatment, especially given the limited long-term safety data and lack of regulatory approval for TMD in many countries.

Similarly, occlusal splints, though widely used, also show heterogeneous outcomes; some randomized trials suggest significant benefits in reducing pain and joint noises, while others find no clear advantage over the placebo or behavioral interventions.

Taken together, these findings reinforce the clinical utility of BTX-A in addressing both pain and functional impairment in TMD patients. Its reversible mechanism, combined with a favorable safety profile and its effects on secondary symptoms such as headache and jaw mobility, makes it a valuable addition to the multidisciplinary management of temporomandibular disorders.

Nevertheless, ongoing research is needed to clarify its indications, optimize dosing protocols, and better understand its effects on joint-related symptoms in comparison to more established modalities like occlusal splints.

### 3.4. Psychological Aspects of TMD Management

The psychological aspects of the treatment of TMD play an important role due to the strong association of these ailments with stress, anxiety, and depressive factors [7,12,16,31,32]. Studies show that psychotherapeutic interventions can be a useful tool in reducing the severity of TMD symptoms.

In a study by Simões and co-authors, both the psychological counseling group and the psychological counseling combined with jaw exercise group showed efficacy in treating TMD, improving patients’ symptoms compared to baseline assessment. The study reported a statistically significant difference in the resolution of TMJ clicks at the end of the follow-up period, with the combined treatment group demonstrating superior outcomes [93].

Digital therapies (DTx) can exert therapeutic effects by controlling behavioral factors through the delivery of appropriate interventions. Park and co-authors conducted an open randomized controlled trial to evaluate the effectiveness of DTx in treating TMD. The RCT demonstrated the potential of DTx in TMD, showing improvements in pain and mouth opening compared to conventional treatment. The intervention group showed a significant reduction in pain scores as measured by the Numerical Rating Scale (NRS) and a statistically significant increase in maximum mouth opening compared to the control group. However, there were no significant changes in other outcomes, as assessed by the Jaw Functional Limitations Scale, the Oral Behavior Checklist, and the Patient Health Questionnaire-4 between the intervention and control [94].

An app has been developed that records TMD pain and parafunctional activities for cognitive behavioral therapy (CBT). However, evidence supporting the reduction in patients’ clinical symptoms through repetitive software-based CBT remains limited. Hwangbo and co-authors conducted a study to investigate the effectiveness of a CBT-supported application in improving clinical symptoms of temporomandibular joint disorders. The intervention group received conservative treatment in combination with app-based therapy, while the control group received conservative treatment only. Compared to the control group, the experimental group showed a significant improvement in the number of tender points and the degree of mouth opening. They also showed an improvement in pain levels, joint sounds, and locking, although these changes were not statistically significant [95] (Table 5).

A pre–post intervention study by de Oliveira Melchior et al. used a mindfulness program to examine sensitivity parameters in women with chronic TMD. It appears that the mindfulness program was effective in reducing parameters suggestive of central pain sensitization in women with chronic, painful TMD. The intervention affected both the stimulus (allodynia, hyperalgesia) and perceptual (PPT) domains of pain sensation. The increase in mindfulness correlated with clinical improvement—suggesting that mental techniques affect the physical experience of pain [96].

Takeuchi-Sato et al. conducted a randomized controlled trial to evaluate the effectiveness of an email-based recording and reminding system for limiting daytime nFTC in patients with TMDs. Thirty patients were randomly assigned to three groups according to the intervention for limiting non-functional tooth contact (nFTC), which are considered etiological factors for temporomandibular disorders. Each group consisted of 10 participants. The first group received cognitive behavioural therapy with an email-based recording and reminding system for 20 days, while the second one received CBT with a sticky note reminder for 20 days, and the third one received simple verbal instructions to avoid non-functional tooth contact. The incidence of nFTC significantly decreased after the intervention in groups one and two. Of the three groups, the decrease in the frequency of nFTC was maximum in group one. In addition, the extent of pain-free, unassisted mouth opening showed a significant increase in all three groups, with the maximum improvement in group one [97].

Studies show that combining psychological therapies with conservative treatment yields better results in reducing pain, improving the mandibular range of motion, and reducing parafunctional behavior (Table 5).

## 4. Discussion

**Figure 1:**
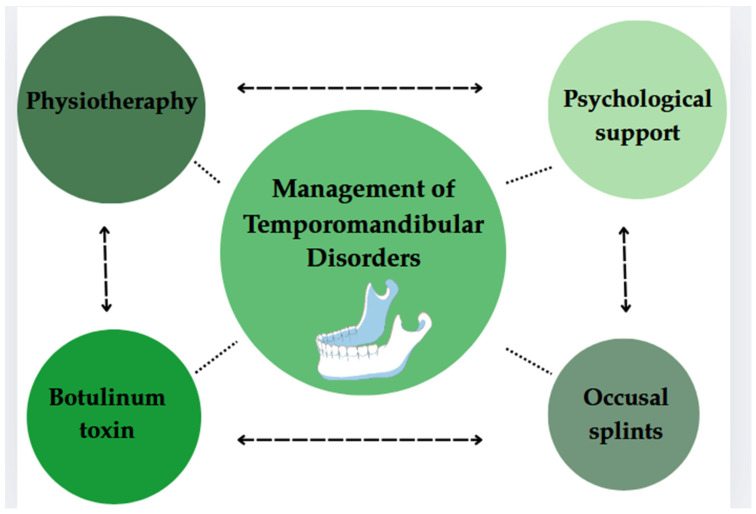
Multidimensional treatment of TMD.

The purpose of this narrative review is to evaluate the effectiveness of a multimodal, multidisciplinary approach in the treatment of temporomandibular disorders (Figure 1).

The management of temporomandibular disorders encompasses a wide range of options, from non-pharmacological conservative therapies to invasive surgical interventions. Although numerous studies have examined treatment strategies for TMD [94,95], the heterogeneity of approaches makes it difficult to establish a universal treatment protocol. Most researchers agree that an effective approach should be multidisciplinary, multiphase, and individualized. A comprehensive therapeutic model that acknowledges the condition’s complexity appears to be the most effective [2,74]. To date, no single therapy has been unequivocally shown to be superior to others.

Physiotherapy is widely recognized as a safe and effective modality in TMD management [80,98,99]. Our findings support its efficacy in alleviating key symptoms such as pain and muscular hyperactivity. Mechanistically, techniques such as mobilization and soft tissue therapy activate proprioceptors and mechanoreceptors, which compete with nociceptive input according to the gate control theory of pain modulation [99]. Functional exercises enhance neuromuscular coordination, restore physiological muscle recruitment patterns, and improve mandibular motor control [100]. Additionally, myofascial techniques facilitate lymphatic and venous drainage in the TMJ region, reducing inflammation and improving tissue trophism [71]. By downregulating sympathetic nervous system activity, physiotherapy creates a physiological state conducive to recovery [101].

However, detailed investigations into the potential adverse effects and long-term durability of physiotherapy outcomes are lacking. The majority of existing studies emphasize short-term efficacy without reporting side effects. Furthermore, the current evidence base is limited by methodological weaknesses, short follow-up durations, and the lack of standardized intervention protocols. Future high-quality clinical trials with extended follow-up periods and clearly defined treatment methodologies are necessary to draw more reliable conclusions [71,102].

Botulinum toxin type A acts by inhibiting acetylcholine release at the neuromuscular junction, resulting in temporary muscle weakening or paralysis. It also modulates pain by suppressing the release of neurotransmitters such as substance P, glutamate, and calcitonin gene-related peptide (CGRP), thereby reducing both peripheral and central sensitization. This mechanism is particularly beneficial in managing chronic muscular pain associated with TMD [103]. The ability of BTX-A to reduce TMJ pain and related headaches, while enhancing jaw mobility, makes it a valuable adjunctive therapy [104]. Injections into the masseter muscle have demonstrated high efficacy and a favorable safety profile, although complications can occur. Peng et al. reported that 30% of subjects experienced transient reductions in masticatory strength, with other side effects including bruising, headaches, smile asymmetry, paradoxical bulging, and hollowing of the cheeks [105]. A systematic review and meta-analysis of both human and animal studies indicated that Botox may cause a reduction in mandibular cortical bone thickness by approximately 6% in humans. Animal models further revealed significant reductions in both cortical and trabecular bone mass. However, the majority of studies did not evaluate the impact of repeated injections or high dosages, highlighting the need for further research into long-term outcomes [106].

This study also demonstrated that occlusal splints increase both resting and functional EMG activity in masticatory muscles, reduce pain, and help restore functional balance within the TMJ and surrounding soft tissues [36,37,80]. Nevertheless, this intervention is not without limitations. A 2010 study documented three cases in which the prolonged use of stabilizing splints resulted in irreversible occlusal changes, including tooth displacement and bite alterations [105]. Albagieh et al. concluded that there is no strong evidence supporting the superiority of occlusal splints over physiotherapy in TMD treatment [37].

Considering the biopsychosocial nature of TMD, a holistic and interdisciplinary approach involving a physiotherapist, dentist, and psychologist often yields the most favorable outcomes, particularly for patients with chronic pain [1]. However, the efficacy of such comprehensive care can vary between individuals, therapeutic effects typically manifest over an extended period, and the overall treatment burden can be high in terms of time and cost. Incorporating psychotherapy into the therapeutic plan may offer substantial benefits but requires careful patient selection and individualized planning [107].

Due to the lack of diagnostic and therapeutic standardization, short follow-up periods, and small sample sizes in many studies, cross-comparison of treatment efficacy remains challenging. Future research should prioritize well-designed randomized clinical trials with larger cohorts, longer observation periods, and greater methodological rigor. Moreover, a deeper understanding of the mechanisms of action underlying various treatment modalities is necessary. We hope that our work contributes to addressing these limitations and encourages a more unified and evidence-based approach to TMD management.

Considering the multifactorial etiology of TMD, a structured yet flexible multimodal framework may optimize treatment outcomes. Based on current evidence, early-stage TMD can be effectively managed with physiotherapy and behavioral strategies, while more chronic or refractory cases may require adjuncts such as BTX-A or occlusal splints. A stepwise, individualized protocol based on the symptom type (muscular, articular, psychosocial), chronicity, and previous treatment response may serve as a clinical decision-making guide.

From a research and clinical perspective, future directions should include the development of standardized multimodal care protocols with consistent outcome measures, such as pain intensity, joint function, and patient-reported quality of life. At the same time, personalization should remain central: factors such as psychological comorbidities, age, or occupational load must inform treatment planning. A stratified approach to care, combined with long-term follow-up and larger randomized studies, may yield clearer insights into optimal treatment combinations and sequencing.

Finally, key clinical recommendations include using physiotherapy as a first-line conservative measure, considering BTX-A only in selected cases due to unresolved safety concerns, avoiding prolonged splint use without regular dental supervision and integrating psychological care where indicated to address chronic pain behavior and emotional distress.

### Limitations

This review is subject to several limitations. First, the included studies exhibit significant methodological heterogeneity in terms of study design, sample size, intervention protocols, and outcome measures, which complicates a direct comparison of results. Second, the lack of standardized diagnostic criteria and treatment protocols for TMD across studies introduces variability that may affect the generalizability of findings. Third, many of the analyzed papers focused on short-term outcomes, with limited follow-up data to assess the durability of treatment effects. Finally, this review did not conduct a formal risk of bias assessment, which limits the ability to critically appraise the overall quality of evidence. A limitation of this study is the exclusion of articles that were not available in open access.

Future systematic reviews should aim to include more homogeneous studies, apply standardized evaluation tools, and assess long-term clinical outcomes to strengthen the reliability of conclusions.

## 5. Conclusions

Temporomandibular disorders are a complex condition influenced by a range of somatic and psychological factors. Due to the wide range of symptoms and the interaction of the temporomandibular joint with the nervous, muscular, and skeletal systems—including the cervical spine—effective management of TMD requires a comprehensive, multidisciplinary approach.

The literature strongly supports conservative treatment as the first-line therapy. This includes manual therapy, kinesitherapy, physical modalities, and the use of occlusal splints. Research shows that properly selected physiotherapy—targeting both the temporomandibular joint and cervical spine—using techniques such as post-isometric relaxation or dry needling, can significantly reduce pain, improve joint function, and enhance patients’ quality of life.

In cases of chronic muscle pain and movement limitations, botulinum toxin type A (BTX-A) may serve as an effective adjunctive treatment. Its analgesic effects and potential to improve mandibular mobility have been confirmed in multiple clinical studies.

In conclusion, TMD treatment should be individually tailored. A multimodal strategy—incorporating physical, psychological, and pharmacological components—offers the most favorable outcomes. Further research is necessary to optimize therapeutic protocols and evaluate the long-term effectiveness of various interventions.

## Figures and Tables

**Table 1:** Inclusion and exclusion criteria.

Data Source	PubMed, Scopus, Google Scholar
**Publication date**	January 2020–2025
**Language**	Open-access English version with full text
**Type of paper**	Clinical trials, pilot study, prospective study, pre–post intervention study, one-group, quasi-experimental, case-series study
**Inclusion criteria**	Articles relating to main focuses with similar materials and methods
**Exclusion criteria**	Non-English-language articles, books, other types of articles No open-access version with full text
**Journal category**	All

**Table 2:** Temporomandibular disorders and physiotherapy.

Authors	TMD and Physiotherapy	Type of Study
Crăciun et al. [64] 2022	Effectiveness of physiotherapy in the treatment of temporomandibular joint dysfunction and the relationship with the cervical spine	Non-randomized controlled
Nambi and Abdelbasset [50] 2020	Efficacy of Maitland joint mobilization technique on pain intensity, mouth opening, functional limitation, kinesiophobia, sleep quality, and quality of life in temporomandibular joint dysfunction following bilateral cervicofacial burns	randomized controlled study
Barone et al. [53] 2021	Immediate effects of rhythmic joint mobilization of the temporomandibular joint on pain, mouth opening, and electromyographic activity in patients with temporomandibular disorders	one-group, quasi-experimental
Gębska et al. [54] 2023	Evaluation of the efficacy of manual soft tissue therapy and therapeutic exercises in patients with pain and limited mobility TMJ	randomized controlled trial
Atilgan et al. [56] 2023	Effect of yoga-based exercise program in female patients with myofascial pain of temporomandibular disorders	randomized controlled trial
Rezaie et al. [63] 2022	Efficacy of neck and temporomandibular joint manual therapy in comparison with a multimodal approach in the patients with TMJ dysfunction	randomized controlled trial
de Oliveira-Souza et al. [65] 2024	Effectiveness of an 8-week neck exercise training on pain, jaw function, and oral health-related quality of life in women with chronic temporomandibular disorders	randomized controlled trial
Dib-Zakkour et al. [66] 2022	Evaluation of the effectiveness of dry needling in the treatment of myogenous temporomandibular joint disorders	randomized controlled trial
Dunning et al. [68] 2024	Dry needling and upper cervical spinal manipulation in patients with temporomandibular disorder: A multi-center, randomized clinical trial	randomized controlled trial
Urbański et al. [69] 2021	Application of manual techniques in masticatory muscles’ relaxation as adjunctive therapy in the treatment of temporomandibular joint disorders	randomized controlled trial
Tariq et al. [70] 2024	Efficacy of massage versus massage with post-isometric relaxation in temporomandibular disorders: A randomized controlled trial	randomized controlled trial
Javed et al. [72] 2024	Comparative effects of post-isometric relaxation technique and Bowen’s therapy on pain, range of motion, and function in patients with temporomandibular joint disorder	randomized controlled trial
Serrano Hernanz et al. [73] 2023	Pressure release technique versus placebo applied to cervical and masticatory muscles in patients with chronic painful myofascial temporomandibular disorder: A randomized clinical trial	randomized controlled trial
Kubala et al. [74] 2022	Multidisciplinary and nonpharmacological management of pain in temporomandibular disorders (TMDs)	randomized controlled trial
Friscia et al. [75] 2024	Pulsed electromagnetic fields (PEMFs) as a valid tool in orthognathic surgery to reduce postoperative pain and swelling: A prospective study	prospective study
Shah et al. [76] 2024	Effectiveness of manual therapy physical therapy in conjunction with patient education for temporomandibular disorders	randomized controlled trial
Aguiar et al. [77] 2023	Education-enhanced conventional care versus conventional care alone for temporomandibular disorders	randomized controlled trial
de Barros Pascoal et al. [78] 2020	Effectiveness of counseling on chronic pain management in patients with temporomandibular disorders	randomized controlled trial
Liu et al. [79] 2025	Effects of patient education on the oral behavior of patients with temporomandibular degenerative joint disease	case-series study

**Table 3:** Temporomandibular disorders and occlusal splints.

Authors	TMD and Occlusal Splints	Type of Study
Gupta et al. [81] 2024	Effect of a centric stabilization splint on masticatory muscles in patients with temporomandibular disorders	pilot study
Bachani et al. [82] 2025	Synergism of occlusal splints along with therapeutic exercise on individuals with temporomandibular joint disorders	pilot study
Wänman and Marklund [83] 2020	Treatment outcome of supervised exercise, home exercise, and bite splint therapy, respectively, in patients with symptomatic disc displacement with reduction	randomized controlled trial

**Table 4:** Temporomandibular disorders and botulinum toxin (BTX-A).

Authors	TMD and BTX-A	Type of Study
Blanco-Rueda et al. [84] 2023	Preliminary findings of the efficacy of botulinum toxin in temporomandibular disorders	pilot study
De La Torre Canales et al. [87] 2022	Long-term effects of a single application of botulinum toxin type A in temporomandibular myofascial pain patients: A controlled clinical trial	randomized controlled trial
Rezazadeh et al. [86] May 2023	Effects of botulinum toxin A injection on the lateral pterygoid muscle in patients with a painful temporomandibular joint click	randomized controlled trial
Kim et al. [89] 2023	Effect of botulinum toxin on masticatory muscle pain in patients with temporomandibular disorders	pilot study
Hosgor et al. [91] 2023	Assessing change in functional outcomes and quality of life in myogenic temporomandibular disorders undergoing botulinum toxin injection: A before and after comparison	randomized controlled trial
De La Torre Canales et al. [92] 2022	Efficacy of botulinum toxin type A I in the improvement of mandibular motion and muscle sensibility in myofascial pain TMD	randomized controlled trial

**Table 5:** Temporomandibular disorders and psychotherapy.

Author	Temporomandibular Disorders and Psychotherapy	Type of Study
Simões et al. [93] 2023	Counselling treatment versus counselling associated with jaw exercises in patients with disc displacement with reduction—a single-blinded, randomized, controlled clinical trial	randomized controlled clinical trial
Park et al. [94] 2024	Randomized controlled trial of digital therapeutics for temporomandibular disorder: A pilot study	randomized controlled trial
Hwangbo et al. [95] 2023	Evaluation of clinical symptoms improvement by cognitive behavioral therapy using a smartphone application in patients with temporomandibular disorder	randomized controlled trial
de Oliveira Melchior et al. [96] 2020	Mindfulness-based intervention reduces sensitivity parameters in women with chronic painful TMD	pre–post intervention study
Takeuchi-Sato et al. [97] 2020	Efficacy of an email-based recording and reminding system for limiting daytime non-functional tooth contact in patients with temporomandibular disorders: A randomized controlled trial	randomized controlled trial
